# Predicting the Impact of Intervention Strategies for Sleeping Sickness in Two High-Endemicity Health Zones of the Democratic Republic of Congo

**DOI:** 10.1371/journal.pntd.0005162

**Published:** 2017-01-05

**Authors:** Kat S. Rock, Steve J. Torr, Crispin Lumbala, Matt J. Keeling

**Affiliations:** 1 Warwick Infectious Disease Epidemiology Research (WIDER), The University of Warwick, Coventry, UK; 2 Life Sciences, The University of Warwick, Coventry, UK; 3 Department of Vector Biology, Liverpool School of Tropical Medicine, Liverpool, UK; 4 Programme National de Lutte contre la Trypanosomiase Humaine Africaine (PNLTHA), Kinshasa, Democratic Republic of Congo; 5 Mathematics Institute, The University of Warwick, Coventry, UK; Foundation for Innovative New Diagnostics (FIND), SWITZERLAND

## Abstract

Two goals have been set for Gambian human African trypanosomiasis (HAT), the first is to achieve elimination as a public health problem in 90% of foci by 2020, and the second is to achieve zero transmission globally by 2030. It remains unclear if certain HAT hotspots could achieve elimination as a public health problem by 2020 and, of greater concern, it appears that current interventions to control HAT in these areas may not be sufficient to achieve zero transmission by 2030. A mathematical model of disease dynamics was used to assess the potential impact of changing the intervention strategy in two high-endemicity health zones of Kwilu province, Democratic Republic of Congo. Six key strategies and twelve variations were considered which covered a range of recruitment strategies for screening and vector control. It was found that effectiveness of HAT screening could be improved by increasing effort to recruit high-risk groups for screening. Furthermore, seven proposed strategies which included vector control were predicted to be sufficient to achieve an incidence of less than 1 reported case per 10,000 people by 2020 in the study region. All vector control strategies simulated reduced transmission enough to meet the 2030 goal, even if vector control was only moderately effective (60% tsetse population reduction). At this level of control the full elimination threshold was expected to be met within six years following the start of the change in strategy and over 6000 additional cases would be averted between 2017 and 2030 compared to current screening alone. It is recommended that a two-pronged strategy including both enhanced active screening and tsetse control is implemented in this region and in other persistent HAT foci to ensure the success of the control programme and meet the 2030 elimination goal for HAT.

## Author Summary

Gambian sleeping sickness is a tsetse-transmitted disease which, without treatment, usually results in death. Unfortunately no medical prophylaxis exists to prevent infection in humans but curative medicines and vector control options are available. Recently there has been a push to reduce disease burden and a target incidence of 1 reported case per 10,000 people per year is hoped to be achieved in 90% of regions by 2020. Subsequently there is a goal of zero transmission by 2030. Using mathematical modelling, we assessed how different intervention strategies such as improving screening and treatment or introducing vector control can help in achieving these goals in a high endemicity setting. Following model simulation, we predict that improving current screening can reduce the time taken until the elimination targets are met. However it is very unlikely that the reported case target will by achieved by 2020 without additional vector control. We found that vector control has great potential to reduce transmission and, even if it is less effective at reducing tsetse numbers as in other regions, the full elimination goal could still be achieved by 2030. We recommend that control programmes use a combined medical and vector control strategy to help combat sleeping sickness.

## Introduction

Gambian sleeping sickness (one of the two forms of human African trypanosomiasis, HAT) has long been a scourge to many in sub-Saharan Africa, with this virulent tsetse-borne disease persisting in numerous foci despite decades of interventions. In recent years, great improvements to curative drugs and screening programmes have contributed to a substantial decline in disease incidence [1]. In addition, advances in tsetse control have meant that control programmes can use strategies with interventions against both the parasite and vector [2, 3].

One such tsetse control method is through the use of insecticidal targets, in particular the recently developed “tiny target” [2, 4]. In areas which have already implemented vector control via tiny targets, a sharp decrease in tsetse abundance has been observed; there has been a reported 80% reduction to tsetse populations in Guinea [3] and 90% reduction in Uganda [2]. To complement regular medical surveillance, such tsetse control has the potential to help push towards elimination of this disease. In 2012, the World Health Organization (WHO), targeted Gambian HAT for elimination as a public health problem (defined as less than 1 reported case per 10,000 per year) in 90% of foci and fewer than 2000 reported cases by 2020. With just over three years left, many foci are thought to be on track to meet this low level of incidence [1, 5]. However some foci of the Democratic Republic of Congo (DRC) are likely to fall in the 10% which do not reach this target level. Whilst these regions will probably not stop achievement of the 2020 goal, which is defined at a global rather than local level, it poses the question of whether changes to intervention strategy could be sufficient to meet the elimination as a public health problem threshold by 2020 in these more challenging areas.

A secondary WHO goal is to completely eliminate HAT, with zero transmission of infection by 2030. Some high-endemicity regions are likely to have transmission remaining at relatively high levels if no change is made to current intervention strategy [6] and they may hinder achievement of this 2030 goal.

In DRC, the former province of Bandundu accounted for 46% of the total cases of HAT between 2000–2012 [7], and so this is a critical region in which the battle against HAT must be won. Using mathematical modelling, a previous study [6] suggested that heterogeneity in risk and behaviour in the human population is an important driver of disease dynamics in the Yasa-Bonga and Mosango health zones of former Bandundu (they are now in the smaller, newly formed Kwilu province following changes to administrative boundaries). In line with social science studies which indicate working adults are underrepresented in screening [8], model comparison of the study indicated that those at highest risk of infection, from highest exposure to tsetse bites, are also less likely to participate in active screening campaigns.

Screening and treatment of the population was estimated to have decreased the incidence of new transmissions by over 50% over a 13-year period [6]. It is now crucial to examine quantitatively how the intervention strategy may be improved to further reduce transmission in Yasa-Bonga and Mosango and ensure that the 2030 WHO elimination goal is met.

### Intervention strategies

The mainstay of HAT control strategy is medical intervention consisting of either passive or active detection. Passive detection occurs when infected individuals self-present to medical facilities following onset of symptoms. Since progression of HAT disease is slow and the initial stage of infection (stage 1 disease) gives rise to relatively mild symptoms, there is a period of around 17 months [9] where individuals are unlikely to seek HAT treatment, or, if they do, the non-specific systems shared with other illnesses including malaria [10] can result in misdiagnosis. During stage 2 disease, defined as the time after the parasite has crossed the blood-brain barrier, patients develop severe symptoms such as behaviour disturbances, lethargy and eventually, without treatment, most cases will result in death [11]. After progressing to stage 2 patients will be more likely to present to medical facilities [12, 13].

In contrast, active detection consists of mobile screening teams travelling to villages and typically testing over 70% of the population. This type of screening can lead to early diagnosis of HAT infection, thereby reducing patient suffering whilst also preventing those patients contributing towards onwards infection. Between 2000 and 2012, annual screening levels in Yasa-Bonga and Mosango ranged from 15% up to 53% of the population and had a mean level of 30%. Initially the reported case incidence lay at 26 cases per 10,000 across the two health zones. In 2009 there was a peak of 40 cases per 10,000, which coincided with the year when screening was greatest. By 2012 there were slightly less than 10 reported cases per 10,000. These data are visualised in Rock *et al*. [6] alongside model fitting results which indicate that underlying transmission incidence is likely to have reduced by 52–53% between 1998 and 2012. Demographic data for the region is sparse; people tend to live in small villages and there is limited information on these populations. Consequently, aggregate data is used in this analysis.

Following case confirmation, one of two treatments is administered; pentamindine for stage 1 disease and nifurtimox and eflorithine combination treatment (NECT) for stage 2. During treatments patients are hospitalised and subsequently are advised to rest at home. The model presented uses a mean duration of 6 months from detection to a return to normal activities and therefore tsetse exposure and the chance to be re-infected by subsequent tsetse bites.

During active screening, the mobile teams attempt to recruit as many of the population as possible, but often certain groups are unrepresented in testing; in particular, adult males, who may work away from the village, are thought to be consistently missed [8]. Another heterogeneity in the human population is exposure to tsetse bites, with individuals spending time in the forested or riverine areas where tsetse concentrate receiving the most bites [14]. A previous modelling study indicated that there was evidence that the highly exposed individuals are also the group which do not participate in active screening [6]. Identifying this key component in transmission and treatment of HAT infection provides an opportunity to improve current active detection strategy by placing emphasis on recruiting individuals in screening who have not previously participated. A similar approach has recently been suggested by Stone *et al*. [15], who deduced that reaching the high-risk individuals should be prioritised to control disease effectively.

Aside from medical intervention, methods to control the tsetse population are available. These include the use of insecticide-treated baits to lure and kill tsetse, or widespread application of insecticides through aerial or ground spraying [16, 17]. Different methods have been shown to be more or less effective in different geographical areas due to factors including the landscape, animal abundance and tsetse species. Unlike other disease vectors such as the mosquito, the unique reproductive biology of tsetse means that it is relatively easy to cause large drops in population sizes by raising mortality just a few percent [18]. In this study the focus is placed on use of tiny targets, which is one method of tsetse control suited to deployment in these health zones. Tiny targets comprise small (0.25 x 0.5 m) panels of insecticide-impregnated blue fabric and black netting which are deployed in the riverine habitats where tsetse vectors of Gambian HAT concentrate. Tsetse are attracted to the target, contact it and, in so doing, pick up a lethal dose of insecticide. Trials in Guinea [3], Kenya and Uganda [2] have shown that deployment of tiny targets can reduce densities of tsetse by 70% to >90%. The targets are cheap (∼US$ 1/target) and easy to deploy, hence control of the tsetse population can be achieved at US$ 84/km^2^ [19]. Shaw *et al*. have assessed the relative costs of other methods (e.g., ground and aerial spraying at US$ 380/km^2^, traps at US$ 283/km^2^) [20] and shown how inexpensive tiny targets are by comparison. Insecticide-treated cattle is also a cheap method (US$ 30/km^2^) [20–22] but this is not possible in areas such as Yasa-Bonga or Mosango where cattle are absent or sparse (<10 animals/km^2^) [23–25].

### Measuring elimination of HAT

In 2012 the WHO set ambitious targets for 10 neglected tropical diseases (NTDs) to be achieved by 2020. For Gambian HAT, this goal is to reach “elimination as a public health problem” which is defined as being less than 1 case per 10,000 people per year in 90% of HAT foci [26]. There is also a global target of fewer than 2000 reported cases per year by 2020. These WHO targets are based upon observed case reporting as this has previously been the only available measure of global or country-level burden of disease in humans. There are, however, caveats in using reported case incidence. Firstly there is thought to be high levels of underreporting, with the WHO previously estimating this at around 65–75% [27, 28]. Secondly, and crucially, the active case detection and reporting is dependent upon both the accuracy of the diagnostic algorithm and the percentage of the population screened.

Rock *et al*. [6] recently proposed an alternative method for evaluating transmission, which is less sensitive to underreporting, screening levels and test sensitivity. This provides an estimate of the incidence of new infections, which can be inferred from reported case data and active screening levels using mathematical modelling. It should be noted that underlying incidence is not the target in the WHO 2020 goals, and that this previous publication did not make the distinction clear.

As might be expected, using observed case reporting, the incidence of HAT infection appears to increase during periods with high levels of screening; for instance high reported incidence was observed in 2009 in Yasa-Bonga and Mosango when the maximum screening (53% of the population) was achieved. Likewise, if improvements are made to combat underreporting of disease, such as using rapid diagnostic tests as part of passive screening in rural locations, this will also result in an increase in reported cases. These two improvements (increased active screening and decreased underreporting) will support a reduction in disease transmission, yet this will not be reflected in reported incidence. The proposed measure of new infection incidence is able to capture the positive impact of such improvements as new infections (transmission) will decrease with the boost in case detection.

### Models for prediction

Mathematical modelling enables the prediction of the possible impact of a range of intervention strategies upon human disease, in addition to providing estimates of underlying transmission and new infection incidence which cannot be directly observed. Using these metrics, predictions can be made for the years in which elimination and elimination as a public health problem can be achieved. Additionally, the number of new cases averted compared to a base-line strategy can be computed.

Previous work by Rock *et al*. [6] which fitted a mathematical model for HAT to human case data from Yasa-Bonga and Mosango has provided estimates for when the incidence of new infections is expected to fall below a threshold of less than 1 new transmission per 10,000 people per year under current interventions. In this region, active screening formed the main component of the intervention strategy and no large-scale tsetse control had yet been implemented. This analysis concluded that using screening alone would not result in swift reduction of infection, and new transmissions would not be likely to drop below 1 per 10,000 until 2059–2091. Consequently these health zones may present problems in the achievement of the 2030 goal of zero transmission.

This article now presents the possible impact of tsetse control, particularly via the use of tiny targets, and improved active screening campaigns in these high endemicity health zones.

## Methods

### Modelling control of tsetse

Modelling the impact of tiny targets on tsetse populations is non-trivial. Rather than simply increasing the mortality of flies, the targets affect flies during the host-seeking phase of their feeding cycle. This has knock-on effects for reproduction, particularly as female tsetse carry their offspring one at a time until ready to pupate.

In this study a mathematical model was developed (for more information, see S1 Model formulation and analysis) which incorporates the following biological aspects of tsetse biology:

- Gamma distributed (*n* = 3) feeding cycles, with a mean time between bites of 3 days.
- Age- and gender-dependent mortality, parameterised using the functional form given by Hargrove [29].
- An explicit pupal stage where the rate of new pupae being deposited is dependent on the number of adult females taking their third feed since they matured or deposited their last offspring. This is to account for the pregnancy period.
- Density dependent tsetse population with growth limited by factors such as parasitism and/or predation on pupae [30].
- Chance of having a lethal contact with a tiny target during feeding phase with a probability of dying dependent upon how long the target was placed in the field. Targets efficacy was assumed to remain high for the first 3 months, followed by a decline due to loss of insecticide. This is similar to the approach taken by other authors [5, 31], but here a sigmoidal function is chosen rather than constant efficacy followed by a linear decline.

Whilst incorporating so much biological complexity results in a more complex mathematical model, the majority of the necessary parameters have been empirically estimated and so the adult emergence rate, and consequently, the “bounce-back” time following target removal, which are unknown can be found from these other parameters. It is also noted in the context of this specific study, that cessation of intervention is not considered, and so the exact mechanism of the “bounce-back” (i.e. population regrowth versus invasion from neighbouring regions) is of less importance.

**Fig 1:**
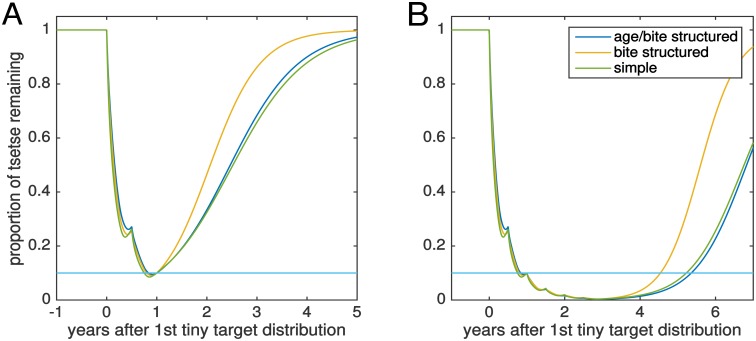
Complex and simple tsetse demography models. The figures compare the complex age- and gender-dependent tsetse model to a simpler version where there has been (A) 1 year and (B) 3 years vector control with 6-monthly target replacement. In this example it is assumed that after one year of targets the population has decreased by 90%. The deposit rate of pupae in the simpler model is chosen so that the “bounce-back” dynamics of the two models are comparable.

A simpler version of this model without tsetse senescence is also used. This version still retains an explicit pupal stage with density dependent mortality and target-dependent deaths only occur with feeding, but the biting pattern is exponentially distributed rather than gamma distributed (see S1 Model formulation and analysis). The simpler version can be parameterised to give similar dynamics (see Fig 1 for a comparison between the structured and unstructured models). It is this simpler model which is then used within a tsetse-host HAT infection model to simulate the impact of tsetse control on human disease dynamics.

### Improvements to screening

The model for tsetse population dynamics in areas where targets are present (described above) was taken in conjunction with an existing model which included active and passive detection and treatment [6]. This new extension allowed the projection of future HAT incidence under various strategies in which existing active screening was combined with vector control. Previous model fitting and selection to observed HAT dynamics in Yasa-Bonga and Mosango between 2000–2012 using the deviance information criterion (DIC) could not conclusively support or rule out the possibility of an animal reservoir in the transmission cycle, and so variants both with and without animal transmission were included here in the analysis (Models 4 and 7). The analysis in the previous article found that the best fitting models had two distinct human subgroups, the first was at low-risk from receiving tsetse bites and participated randomly in active screening, whereas the second were both at high-risk and had systematic non-participation.

The analysis presented here assessed the impact of changing active screening strategy to focus on being able to recruit high-risk individuals to the screening programme. This was done so that three active screening strategies could be evaluated: (i) only low-risk individuals are actively screened (as in [6]) (ii) both low- and high-risk individuals had the same probability of participating in screening (iii) the high-risk population were targeted for screening with the remaining screening occurring randomly from the low-risk population. It is assumed that this change in screening occurs from 2017 onwards.

### Combined strategies

The key strategies consisted of:

- Actively screening 30% of the human population from 2017 onwards, this corresponds with the mean level achieved between 2000 and 2012 in the region [1, 32]. For the years 2013–2015, the level of active screening is known (although the number of cases is not).
- Recruiting people to active screening with either (a) low-risk only (the current situation), (b) both low- and high-risk equally, or (c) all those at high-risk and the remainder at low-risk of exposure to tsetse bites.
- Implementing (a) no vector control, or (b) vector control beginning in 2017. For the strategies with tsetse control, 60% reduction in tsetse density after 1 year was assumed (described here as moderately effective).

These six key strategies are given in Table 1. In addition to these key strategies, variations upon these include:

- higher screening coverage at the maximum level (53%) achieved between 2000–2012
- vector control beginning in 2018
- 90% reduction in tsetse density after 1 year (described here as highly effective).

**Table 1:** Key intervention strategies under consideration.

	Recruited to screening			Tsetse reduction	
Strategy name	Low-risk only	Equal	High-risk first	0%	60%
Screen low-risk	X			X	
Screen equally		X		X	
Screen high-risk			X	X	
Screen low-risk + VC	X				X
Screen equally + VC		X			X
Screen high-risk + VC			X		X

This table presents the proposed screening and vector control strategies. For strategies with vector control it is assumed that initial target deployment begins in 2017 and achieves 60% reduction in tsetse density after one year. The amount of annual active screening assumed is 29.9%, which is the mean level achieved between 2000–2012. Other variations on these strategies are presented in the SI.

These additional interventions, resulting in 18 different strategies are given in Table S3 in S1 Model formulation and analysis.

As mentioned previously, the specificity of diagnostics will impact the reported number of cases. At present the diagnostic algorithm consisting of the card agglutination test for trypanosomes (CATT) followed by subsequent parasitological confirmation has a high, but imperfect specificity, with the value here taken to be 99.9% [33]. Such a specificity would result in 5 detections per 10,000 uninfected people if 50% of the population were screened and so it is likely that in this setting some reported cases are false positive detections. However as the number of detections fall, suspected cases are more carefully scrutinised and other methods can be utilised to increase specificity to 100%.

To account for this improvement in case detection specificity as the reported active and passive cases decline, the model uses the assumption that specificity switches from 99.9% to 100% at some critical threshold. Ultimately this threshold could be chosen in a variety of different ways; here it is taken to be once there are fewer than 2 reported cases per 10,000 above the expected incidence of false positives based on the the level of screening. For example, if 40% of the population were screened in 2019, then the expected incidence of false positives (for an uninfected population) would be 4 per 10,000. Therefore if there were fewer than 6 reported cases per 10,000 in 2019, this would fall below the threshold and so in 2020 the specificity would be increased to 100%. For Yasa-Bonga and Mosango, if there were a similar incidence (6 cases per 10,000 found under 99.9% specificity testing) and also 40% screening in 2020 this would equate to approximately 70 HAT suspects which required more rigorous testing.

The full ODE model equations and fixed parameters, such as population size, tsetse bite rates and disease durations used for this analysis are given in S1 Model formulation and analysis. A compartmental model diagram can also be found in S1 Model formulation and analysis. Parameters which could not easily be established from empirical data, included: the tsetse to human ratio, relative exposure of tsetse bites for different groups of the population and the level of underreporting. These were previously fitted [6] and the posterior parameters distributions found in that study were used here to generate samples for the intervention evaluation. As with the previous analysis, the performed simulations computed the continuous underlying host-vector disease dynamics which was then converted to an annual reporting incidence of passive and active detections, and new infections. The new infection incidence in humans was computed for the strategies under consideration, allowing the expected year of reaching less than 1 new infection per 100,000 people to be estimated. As this threshold is extremely low, equating to fewer than 3 cases in total across both health zones, and so is used as a proxy for full elimination and achievement of the 2030 goal. Additionally, the projected reported number of cases was compared with the elimination as a public health problem threshold to identify whether these health zones could achieve this goal for the different strategies.

## Results

**Fig 2:**
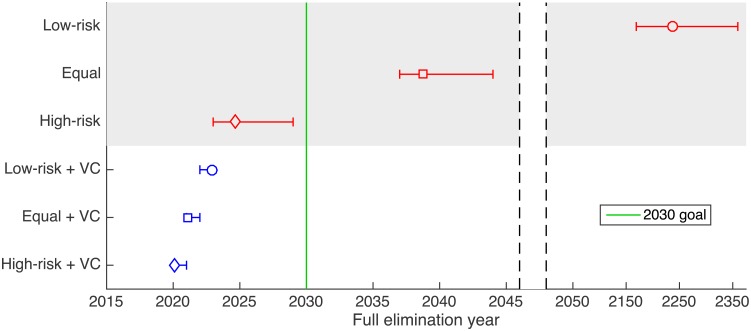
Impact of possible strategies on elimination. The figure shows the predicted year in which transmission drops below 1 case per 100,000 under the six different strategies and compares this to the target year of 2030.

**Fig 3:**
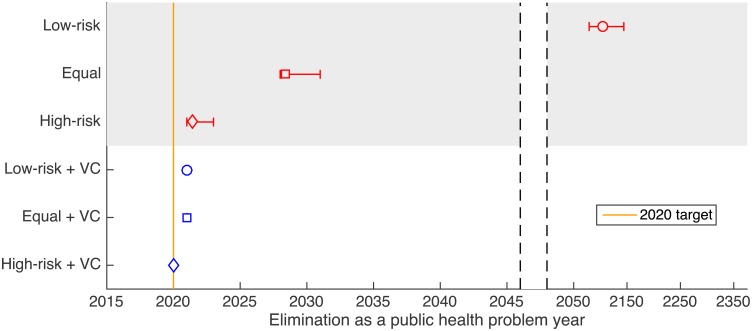
Impact of possible strategies on elimination as a public health problem. The figure shows the predicted year in which the reported cases drop below 1 case per 10,000 under the six different strategies. The results are compared to the year 2020 to see if it is possible that these regions could be amongst the foci that achieve the elimination as a public health problem target within this timeframe.

Figs 2 and 3 give the elimination year results of simulations under each of the key strategies. Further results using variations on key strategies are shown in Figs. S5 and S6 and Tables S4 and S5 in S1 Model formulation and analysis. The total number of cases averted between 2017 (the start of new strategies) and 2030 compared to screening low-risk people alone is presented in Table S6 in S1 Model formulation and analysis. It is noted that the inclusion of animal reservoirs made little difference to the results with 95% credible intervals for models with and without a non-human animal reservoir largely overlapping.

### Impact of intervention strategies on transmission

It is very unlikely that the 2030 full elimination target will be met by continuing with strategies which screen only low-risk members of the population and with no vector control (Fig 2). The predicted elimination year is 2236 for mean screening, and 2121 for maximum screening. This demonstrates how the current strategy is unlikely to impact HAT transmission sufficiently to reach full elimination by 2030.

However, in the absence of vector control, improving screening so that all people are screened equally has a huge impact on the expected year in which the transmission will drop below 1 per 100,000. With maximum screening levels the time is reduced by 94 years (in the year 2027) and with mean screening levels, by 198 years (in the year 2039).

Targeting high-risk people in recruitment, so that all high-risk people are screened and the remaining screened are low-risk, reduces the time to the target transmission threshold even further. Even a modest screening level (the mean achieved between 2000–2012) would likely be sufficient to meet the 2030 elimination target.

With large-scale reductions in vector density of either 60% or 90% there was a dramatic reduction upon transmission of disease and, under any of the strategies (7–18), the 2030 elimination goal was predicted to be met in or before 2024. If a modest 60% reduction was achieved then changing screening strategy meant that the elimination year was bought further forwards and was dependent upon the level and type of recruitment used for active screening. Changing screening from low-risk only to everyone equally bought the elimination date forward by around two years. In simulations of vector control with a 90% reduction in tsetse density across the area, changing screening strategy from current (low-risk only) to strategies which screen all people equally or high-risk first did not substantially change the elimination year; in both cases this occurred after just 1–2 years after tiny targets deployment. With this vector reduction there are only small differences between using mean and maximum screening levels. Finally, with a combination of high-efficacy vector control and recruiting all high-risk individuals to screening, the new infection incidence could be reduced to below the elimination threshold after just one year of implementation.

The number of cases averted (Table S6 in S1 Model formulation and analysis) follows the same trend; changes that make the most difference are either recruiting some high-risk groups to active screening and/or introducing vector control. Whilst increasing the percentage tsetse reduction from 60% to 90% could make almost four years difference to the full elimination year if only low-risk people are screened, there is a relatively minor increase in the number of cases averted if high rather than moderate tsetse control is achieved. All vector control strategies are expected to result in over 6200 averted cases compared to mean screening alone and equal or high-risk screening without vector control is predicted to avert over 4900 cases. By comparison, increasing the level of screening from 30% to 53% is expected to only avert 1410 cases.

### Impact of intervention strategies on reported cases

Qualitatively similar results are obtained for the elimination as a public health problem year, which is when reported cases reached less than 1 case per 10,000 (Fig 3). Under current strategies with either mean or maximum screening (strategies 1 and 2), there was a substantial delay to the time elimination as a public health problem was achieved.

Strategies which improved screening but remained without vector control (Strategies 3–6) greatly reduced the time until elimination as a public health problem but failed to meet it by 2020. All strategies with vector control (7–18) were predicted to meet the elimination as a public health problem threshold by 2022 and 7 vector control strategies (all starting in 2017) were found to be sufficient to achieve the target in 2020 if 90% vector control was achieved.

### Relationship between transmission and reporting

**Fig 4:**
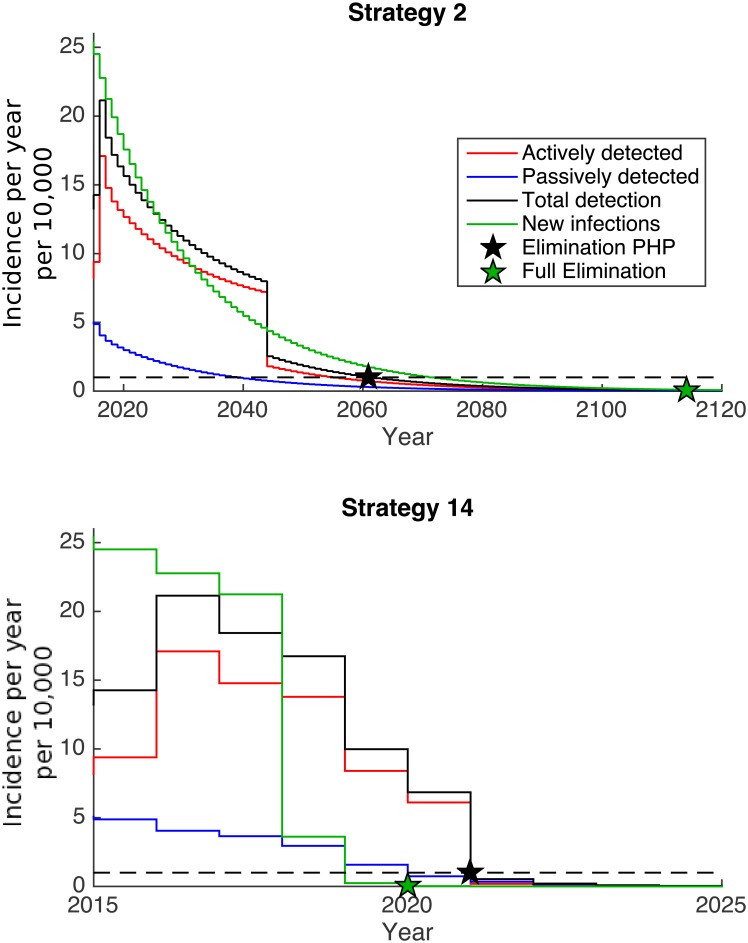
Examples of the relationship between transmission and reporting. The figures present two example simulations showing transmission (green lines) and reporting (black lines). Total reporting consists of both active and passive detections. In the case where transmission is reduced slowly through detection and treatment alone (Strategy 2), transmission remains above the reported cases (since not all the population are screened and there is also underreporting). Conversely, with high efficacy vector control (Strategy 14), transmission drops sharply due to the sudden reduction in tsetse numbers, however the previously infected individuals will continue to be detected both in active and passive screening for several years.

There is a complex relationship between transmission and reporting. There is the trend that the strategies that give the best (or worst) outcomes for full elimination are the same as those that give the best (or worst) results for elimination as a public health problem. In the absence of vector control, elimination as a public health problem is predicted to be achieved before full elimination as naturally expected. In contrast, for many strategies with vector control, transmission will fall below 1 new transmission per 100,000 in advance of reported cases dropping below 1 case per 10,000. For these strategies, the level of vector control achieved was sufficient to halt transmission quickly and whilst there was a consequent rapid drop in new cases, previously infected individuals would still remain infected until they were detected and treated, which could take several years given the disease progression and screening intensity for HAT. Two examples of reported cases compared to transmission are shown in Fig 4.

### Discussion

Modelling predicts that whilst the increasing the level of active screening achieved and improving efficacy of vector control contribute to a decrease in transmission and time to elimination, most gains are made either by (a) improving recruitment high-risk people to screening or, (b) adding some vector control. The results here highlight the great potential for vector control in DRC and these mirror the type of outcomes which have been observed in other areas; the Boffa area of Guinea currently appears on track to meet the target of less than 1 case per 10,000 [5] following introduction of vector control strategies.

This study does not consider the financial implications of different strategies, only projected timeline until the elimination target is met. However, in another modelling study which does consider cost-effectiveness of vector control and medical intervention [31], strategies including vector control are found to be the most cost-effective—due to the low cost of tiny targets. The dramatic results for vector control presented here suggest that the reduction in the length of time for which interventions are needed may mean that strategies with tsetse intervention are more cost-effective, but further work is needed to determine this explicitly. There will be a balance between the costs of tsetse control and its impact; for example is achieving 90% vector control through six-monthly target deployment more or less cost-effective than a lower level of tsetse control achieved through annual deployment? It would also be beneficial to consider the cost-effectiveness of other methods of vector control. Studies conducted on small islands in Lake Victoria, Kenya, targets deployed at 50–100 m intervals along the shore were estimated to impose a daily mortality of 3–6% and targets deployed at 50 m intervals along rivers in the West Nile region of Uganda, giving overall densities of ∼6 targets/km^2^ in, were estimated to impose a daily mortality of 4%. Costs of deploying targets at densities of 6 targets/km^2^ to impose a daily mortality of 4% were estimated at USD 84/km^2^ [2, 19] which gives some indication of the potential costs associated with adding a tiny target strategy to current medical interventions.

Another possible change to intervention strategy is to reduce the high level of underreporting by improving passive case detection. In some regions including Chad [34], Uganda [35] and the Kongo Central (formally Bas-Congo) province of DRC [36], efforts have already been made to increase access to HAT diagnostics by ensuring more local medical facilities carry rapid diagnostic tests (RDTs). Due to uncertainty in the way such schemes may affect passive reporting this was not incorporated in the study presented here, but the doubtless benefits of improving access to HAT diagnosis should not be overlooked.

In the future, new developments in HAT drugs could change the way in which interventions are carried out. Two promising oral drugs, fexinidazole and oxaborole, are in the pipeline with the aim of improving the administration of HAT treatment [37]. It is hoped that both drugs will be stage-independent, removing the need for a lumbar puncture in the diagnostic algorithm, and ideally that they have a suitable safety profile which would allow for much simpler test and treat strategy, potentially just using an RDT [38]. Such a strategy could result in much faster time to treatment by reducing the time spent performing multiple diagnostic tests in the field for active screening, or finding health facilities with the ability to perform the necessary tests to confirm a HAT case for passive detection. Finally, drugs which could be administered on an outpatient basis could encourage screening uptake as hospital admission has previously been identified as one barrier [39].

The structure of the model presented here and in Rock *et al*. [6] captures key components of our current understanding of HAT transmission dynamics with unknown parameters that can be inferred from the available data. This type of model structure is relatively flexible to new additions, such as the tsetse control part of the model that was developed for this study. In future work it will also be necessary for other elements to be incorporated as new technologies alter the diagnostic and treatment options available.

This modelling work necessarily makes many assumptions and these should be taken into consideration with the results presented here. Firstly, this model has not been validated to additional data from these health zones. Once later data (e.g. 2013-2015) becomes available, the predictive ability of the model in this region can be assessed by comparing predicted and actual reported cases. Secondly, the effectiveness of vector control in this region is yet to be assessed and the level and type of screening strategy achieved is unlikely to exactly match those described. Despite this, the selected strategies should be representative of the type of results that could be anticipated.

Additionally, there are spatial heterogeneities which have not been taken into account. For example, it is more likely that people living near larger settlements take part in screening and that there will be variation in tsetse abundance across different regions. Instead a homogeneous assumption was made, due to lack of knowledge on population movements and timing of screening in different areas. The resolution used in this analysis by utilising aggregate data is intended to provide a policy guide to measure the overall progress towards the elimination goals in these areas, rather than make higher-resolution intervention planning decisions, for example, where to place tiny targets or which villages to screen. Modelling could certainly provide more detailed evaluations of that type as well as evaluate how spatial heterogeneities could influence elimination but novel stochastic models with a much greater degree of complexity would be required.

The data provided were based on annual reporting and so active screening was assumed to take place at the beginning of each year. This was the most reasonable parsimonious assumption given the data. Likewise the timing of the initial vector intervention was taken to coincide with active screening. It was assumed that all targets are deployed rapidly, although it is acknowledged here that it could take several weeks to cover the health zones. This could impact predicted outcomes, but since the tsetse density is matched to reductions similar to those observed in the field at 1 year post-intervention. It is believed that any initial discrepancies will not greatly affect the model outcomes which are presented yearly, although it is acknowledged this could impact upon accuracy of predictions, particularly in the short-term. Improved temporal resolution of, say, month of active and passive detections, the length of time needed to deploy targets, and the timing between screening and target placement, would help to give greater confidence in the estimates, particularly in the short-term. Nonetheless it is thought that long-term predictions will remain qualitatively unchanged.

Under this model, an assumption was made that stage 1 individuals would not be detected through the passive system. This modelling decision was taken as most passive detections are made in stage 2, and the data did not contain staging information to infer the passive detection rate of stage 1 cases. In future work, the addition of stage 1 passive detection will become more important as the consequences of improving the passive surveillance system—through increasing the number of health facilities with HAT diagnostics—will be examined.

In this analysis it was assumed that the current diagnostic algorithm specificity could increase from 99.9% to 100% following extra validation of cases at very low incidence. The trypanolysis test may provide one way to achieve 100% specificity, although the test currently requires samples to be sent away for analysis, meaning that it has primarily been used during field trials rather than as standard procedure [40]. Improved specificity explains how other regions with active detection campaigns can report no cases [1]. On-going work fitting the model to data from other regions of DRC shows that in many low-endemicity areas 100% specificity is indeed a better fit to the data than 99.9%.

Under the model this improvement to screening is assumed to happen once incidence reaches low levels. For simplicity and due to lack of available data, we have assumed a step-change for this improvement; a more gradual change would likely delay elimination as a public health problem by a few years. It is stressed that, without some change to specificity of the diagnostic algorithm, it would be unlikely to achieve less than 1 reported case per 10,000 whilst active screening continues at moderate levels, due to the number of false positives detected. Imperfect specificity would not impact on the transmission dynamics themselves and so full elimination predictions would not change.

Yasa-Bonga and Mosango represent just two health zones from former Bandundu province, therefore this study cannot explicitly address the achievability of the 2020 goals as these are defined at a global rather than local level. In future analysis it will be important to perform similar modelling studies to assess the impact of alternative intervention across the whole of this province which has a comparatively high HAT burden, and also in other regions in which HAT infection persists. The study here utilised previous model fitting of the effective tsetse-host ratio in Yasa-Bonga and Mosango to predict the impact of moderate and effective tsetse control for this area. It should be noted that the pre-intervention host-vector ratio and other parameters associated with the basic reproductive ratio will be an important factor in prediction for other areas.

Other future studies should consider the timescale for which interventions must be implemented before they can be lifted without recrudescence occurring. Whilst the threshold of less than 1 case per 100,000 is used as a proxy for full elimination, future elimination/recrudescence studies will likely utilise stochastic models which can incorporate chance events that become important at low numbers of infections. This type of study is also made more complex by having to consider the mechanisms by which tsetse populations may reestablish following removal of tiny targets. For example if there are no flies left in one area, reinvasion from neighbouring regions which did not use tsetse control will be the only way for tsetse populations to recover. If tsetse control merely suppressed the number of flies, bounce-back will be due to a combination of new births and importations. Once appropriate reinvasion models have been formulated, they could also examine the impact of tsetse control on a small scale, which could be useful in the case that some regions achieve elimination whilst others still have HAT infection.

Finally, this study presents a range of intervention strategies which are deemed to be possible to achieve, but some will inevitably be easier to implement than others. In particular it is not clear exactly how the posed improvements to screening may be achieved. More high-risk people (those receiving the most tsetse bites) could potentially be recruited by moving the locations of screenings closer to their daily activities, or altering the time of screening so that they are not away from the village. Mpanya *et al*. [13] suggest that education is essential to improve screening participation as there are often taboos surrounding diagnosis and misinformation about treatment regimes. This may be one focus of improving screening participation and it will be vital to learn from social science to understand how best this issue can be tackled. Undeniably, one of the reasons these regions have not previous implemented vector control is the complex task of placing targets in such a remote and rural locations and this challenge should not be disregarded. Strategy planning should find the best way to combine medical and vector interventions taking into account intervention effectiveness, cost and practicality.

## Supporting Information

S1 Model formulation and analysis Model formulation and analysis. Detailed model description and equations for vector control and HAT infection dynamics are given. Additional results are presented.
(PDF)

S1 Model Code Model code. MATLAB code used for the forward projections of HAT infection.
(M) Click here for additional data file.
