# Supplementary material for: Predicting the Impact of Intervention Strategies for Sleeping Sickness in Two High-Endemicity Health Zones of the Democratic Republic of Congo
Source: PLoS Negl Trop Dis. 2017 Jan 5;11(1):e0005162. doi: 10.1371/journal.pntd.0005162 (PMC5215767; doi:10.1371/journal.pntd.0005162)
Supplement: S1 Model formulation and analysis — Detailed model description and equations for vector control and HAT infection dynamics are given. Additional results are presented. (PDF) [file pntd.0005162.s001.pdf]

# Supplementary Information
# Predicting the impact of intervention strategies for sleeping sickness in two high-endemicity health zones of the Democratic Republic of Congo


Kat S. Rock*[1,2], Steve J. Torr[2,3], Crispin Lumbala[4],Matt J. Keeling[1,2,5]

* Corresponding author: k.s.rock@warwick.ac.uk
1 Life Sciences, Warwick University, Coventry, CV4 7AL, UK
2 WIDER, Warwick University, Coventry, CV4 7AL, UK
3 Liverpool School of Tropical Medicine, Liverpool, L3 5QA, UK
4 Programme National de Lutte contre la Trypanosomiase Humaine Africaine (PNLTHA), Kinshasa, Democratic Republic of Congo
5 Mathematics Institute, Warwick University, Coventry, CV4 7AL, UK


## S1 Model formulation

### S1.1 Vector-only models

Two tsetse-only models where developed in this analysis: the first "detailed tsetse model" (S1.1) includes many aspects of vector behaviour and biology, whereas the second "simplified tsetse model" (S1.2) has less complexity whilst retaining features (for example teneral status) which are deemed essential for appropriately modelling tsetse when used in a host-vector model with disease.

The development of the detailed model, for which parameters can be found in the literature, allowed for the parameterisation of the simplified model. In particular, the per capita rate of new pupal deposits, $B_V$, could not be directly drawn from known parameters. The parameter $B_V$, which only occurs in the simple model, was chosen such that the simplified model closely matched the dynamics of the detailed model following target placement.

The detailed tsetse model includes:

- a distinction between male and female flies, with female flies having longer life expectancy and giving birth to/depositing the next generation of pupae
- senescence/age-dependent mortality using number of bites taken as a proxy for age (including increased teneral mortality and unsuccessful production of pupae)
- increased teneral feeding rate (i.e. the time from emergence to the first feed is shorter than time between subsequent successive feeds)
- gamma distributed ($n = 3$) pupal stage
- gamma distributed ($n = 3$) time between feeds (instead of exponentially distributed)
- a requirement of 3 feeds before each pupa is deposited by a female fly
- tiny-target dependant mortality occurring whilst the flies are host-seeking

- female sexual maturity after first blood-meal

The detailed tsetse model is given by equations (S1.1) with parameterisation explained in Table S1. Each class/tsetse stage has several corresponding equations. For example, female pupae are given by $P_{f1}, P_{f2}$ and $P_{f3}$. Likewise there are 3 teneral stage equations for both male and females and many non-teneral equations so that mortality is allowed to change with age. Female tsetse produce a single pupa on every third blood-meal which corresponds to $G_{f9i}$ where $i \in \mathbb{Z}$. All biting flies, which are contained in $S_{f3}, S_{m3}, G_{f3i}, G_{m3i}$ with $i \in \mathbb{Z}$, have a risk of target-induced mortality given by the function $f_T(t)$.

Detailed tsetse model (no disease):

Female

Pupal

$$\begin{aligned}
\frac{\mathrm{d}P_{f1}}{\mathrm{d}t} &= \tfrac{1}{2}3\alpha(1-f_T(t))\textstyle\sum_{i=1}G_{f9i} - (3\xi_V + \frac{\sum_i(P_{fi}+P_{mi})}{K})P_{f1} \\
\frac{\mathrm{d}P_{f2}}{\mathrm{d}t} &= 3\xi_V P_{f1} - (3\xi_V + \tfrac{\sum_i(P_{fi}+P_{mi})}{K})P_{f2} \\
\frac{\mathrm{d}P_{f3}}{\mathrm{d}t} &= 3\xi_V P_{f2} - (3\xi_V + \tfrac{\sum_i(P_{fi}+P_{mi})}{K})P_{f3}
\end{aligned}$$

Teneral

$$\begin{aligned}
\frac{\mathrm{d}S_{f1}}{\mathrm{d}t} &= 3\xi_V \mathbb{P}(\text{survive pupal stage})P_{f3} - (6\alpha + \mu_f(0))S_{f1} \\
\frac{\mathrm{d}S_{f2}}{\mathrm{d}t} &= 6\alpha S_{f1} - (6\alpha + \mu_f(0.5))S_{f2} \\
\frac{\mathrm{d}S_{f3}}{\mathrm{d}t} &= 6\alpha S_{f2} - (6\alpha + \mu_f(1))S_{f3}
\end{aligned}$$

Non-teneral

$$\begin{aligned}
\frac{\mathrm{d}G_{f1}}{\mathrm{d}t} &= 6\alpha S_{f3}\big(1-f_T(t)\big)S_{f3} - (3\alpha - \mu_f(1.5))G_{f1} \\
\frac{\mathrm{d}G_{fi}}{\mathrm{d}t} &= 3\alpha G_{f(i-1)} - (3\alpha - \mu_f(0.5+i))G_{fi} \\
&\qquad (i \in 3\mathbb{Z}-1, 3\mathbb{Z}-2) \\
\frac{\mathrm{d}G_{fj}}{\mathrm{d}t} &= 3\alpha\big(1-f_T(t)\big)G_{f(j-1)} - (3\alpha - \mu_f(0.5+j))G_{fj} \\
&\qquad (j \in 3\mathbb{Z})
\end{aligned}$$

Male

Pupal

$$\begin{aligned}
\frac{\mathrm{d}P_{m1}}{\mathrm{d}t} &= \tfrac{1}{2}3\alpha(1-f_T(t))\textstyle\sum_{i=1}G_{f9i} - (3\xi_V + \frac{\sum_i(P_{fi}+P_{mi})}{K})P_{m1} \\
\frac{\mathrm{d}P_{f2}}{\mathrm{d}t} &= 3\xi_V P_{m1} - (3\xi_V + \tfrac{\sum_i(P_{fi}+P_{mi})}{K})P_{m2} \\
\frac{\mathrm{d}P_{m3}}{\mathrm{d}t} &= 3\xi_V P_{m2} - (3\xi_V + \tfrac{\sum_i(P_{fi}+P_{mi})}{K})P_{m3}
\end{aligned}$$

Teneral

$$\begin{aligned}
\frac{\mathrm{d}S_{m1}}{\mathrm{d}t} &= 3\xi_V \mathbb{P}(\text{survive pupal stage})P_{m3} - (6\alpha + \mu_m(0))S_{m1} \\
\frac{\mathrm{d}S_{m2}}{\mathrm{d}t} &= 6\alpha S_{m1} - (6\alpha + \mu_m(0.5))S_{m2} \\
\frac{\mathrm{d}S_{m3}}{\mathrm{d}t} &= 6\alpha S_{m2} - (6\alpha + \mu_m(1))S_{m3}
\end{aligned}$$

Non-teneral

$$\begin{aligned}
\frac{\mathrm{d}G_{m1}}{\mathrm{d}t} &= 6\alpha S_{m3}\big(1-f_T(t)\big)S_{m3} - (3\alpha - \mu_m(1.5))G_{m1} \\
\frac{\mathrm{d}G_{mi}}{\mathrm{d}t} &= 3\alpha G_{m(i-1)} - (3\alpha - \mu_m(0.5+i))G_{mi} \\
&\qquad (i \in 3\mathbb{Z}-1, 3\mathbb{Z}-2) \\
\frac{\mathrm{d}G_{mj}}{\mathrm{d}t} &= 3\alpha\big(1-f_T(t)\big)G_{m(j-1)} - (3\alpha - \mu_m(0.5+j))G_{mj} \\
&\qquad (j \in 3\mathbb{Z})
\end{aligned}$$

(S1.1)

Simple tsetse model (no disease):

$$\begin{aligned}
\text{Pupal} \quad & \frac{\mathrm{d}P_V}{\mathrm{d}t} &=& \ B_V N_V - (\xi_V + \tfrac{P_V}{K})P_V \\
\text{Teneral} \quad & \frac{\mathrm{d}S_V}{\mathrm{d}t} &=& \ \xi_V \mathbb{P}(\text{survive pupal stage}) P_V - \alpha S_V - \mu_V S_V \\
\text{Non-teneral} \quad & \frac{\mathrm{d}G_V}{\mathrm{d}t} &=& \ \alpha\big(1 - f_T(t)\big) S_V - \alpha f_T(t) G_V - \mu_V G_V
\end{aligned} \tag{S1.2}$$

Table S1: **Tsetse-only model parameters**

| **Parameter** | **Description** | **Assumed value** |
|---|---|---|
| $\mu_f(a), \mu_m(a)$ | female and male tsetse mortality at age $a$ since emergence respectively | see below (S1.3) |
| $\mu_V$ | combined mortality (across ages and sexes) in simplified model | 0.03 days $^{-1}$ |
| $K$ | pupal density dependence term | 11.09$\times N_{V0}$ |
| $\alpha$ | bite rate | 0.333 days $^{-1}$ |
| $B_V$ | total deposit rate in simplified model | 0.0505 days $^{-1}$ |
| $\xi_f, \xi_m$ | pupal death rate for males and females respectively | (0.038,0.036) days $^{-1}$ |
| $\xi_V$ | combined pupal death rate (across sexes) in simplified model | 0.037 days $^{-1}$ |
| $\mathbb{P}$(survive pupal stage) | probability of pupating | 0.75 |
| $f_T(t)$ | probability of both hitting a tiny target and subsequently dying at time $t$ | see below (S1.4) |

The tsetse mortality functions for males and females take the form:

$$\mu_{f/m}(a) = k_1(k_2 \exp(-k_2 a) + k_3 \exp(k_3 a)) \tag{S1.3}$$

where $(k_1, k_2, k_3) = (0.605, 0.201, 0.0119)$ for females and $(k_1, k_2, k_3) = (0.389, 0.395, 0.0583)$ as first given by Hargrove [S8].

## Impact of tiny targets on tsetse

Fig. S2 shows the dynamics of tsetse populations (under the simple model) where targets are either moderately effective, with a 60% reduction (lower than in Guinea), or highly effective, with a 90% population density reduction in a year (as seen in Uganda).

The function which describes the probability of both hitting a target and dying is time dependent (days) from when the targets where placed:

$$f_T(t) = f_{\text{max}} \left(1 - \frac{1}{1 + \exp(-0.068(mod(t, 182.5) - 127.75))}\right) \tag{S1.4}$$

and $f_{\text{max}}$ is chosen such that the tsetse population after one year is at the observed/assumed percentage reduction. For the simplified model this is given by $f_{\text{max}} = 0.0302$ for a 60% reduction and $f_{\text{max}} = 0.0748$ for a 90% reduction.

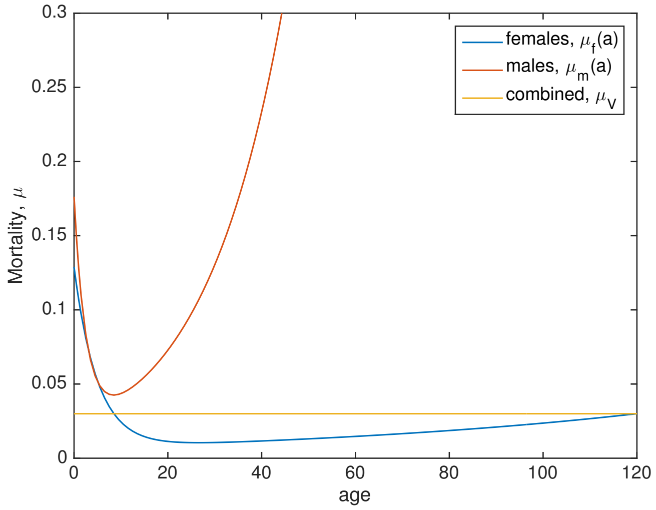


Figure S1: Age-dependent mortality used in the detailed tsetse-only model in comparison to the constant, age-independent mortality assumed in the simplified model. Age-dependent mortality was parameterised using values from Hargrove [S8]

.

## S1.2 HAT infection model

The HAT model equations are given below (S1.5) and correspond with Fig S3. The model is largely the same as that presented by Rock *et al* [S11] but with novel tsetse equations which correspond to the simplified tsetse-only model (S1.2) with additional infection. In the absence of tsetse control, the equations are equivalent to the previous model.

Human hosts are assumed to be in one of four distinct classes: either low-risk and randomly participate in screening (subscript $H1$), high-risk and random participation ($H2$), low-risk and never participate in screening ($H3$) or high-risk and never participate. Tsetse bites are assumed to be taken on humans or animals. The model incorporates reservoir animals which can become infected and assumes that the remainder of the bites are taken on non-reservoir animal species which do not need to be explicitly modelled.

All parameters match those given in Table 1 [S11], additionally $p_V = 0.065$ and $\varepsilon = 0.05$. The free parameters, $k_1, k_2, k_3, k_4, m_{eff}, r, f_A, N_A, u$ (denoted by "Varies" in the table) were previously fitted to data using MCMC and in this analysis take these fitted values.

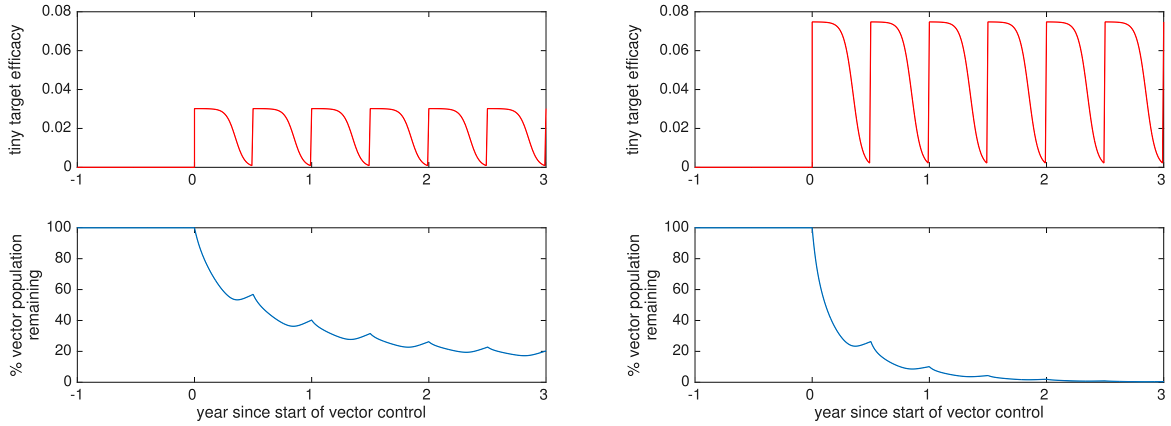


Figure S2: **Impact of tiny targets on tsetse density.** The figures show the how varying target efficacy (red line) impacts tsetse population density (blue line). Target efficacy is measured as the proportion of a host-seeking tsetse which will both hit the tiny target and die as a result. The graphs show the necessary efficacy of targets needed to reduce density by (a) 60% and (b) 90% by the end of the first year.

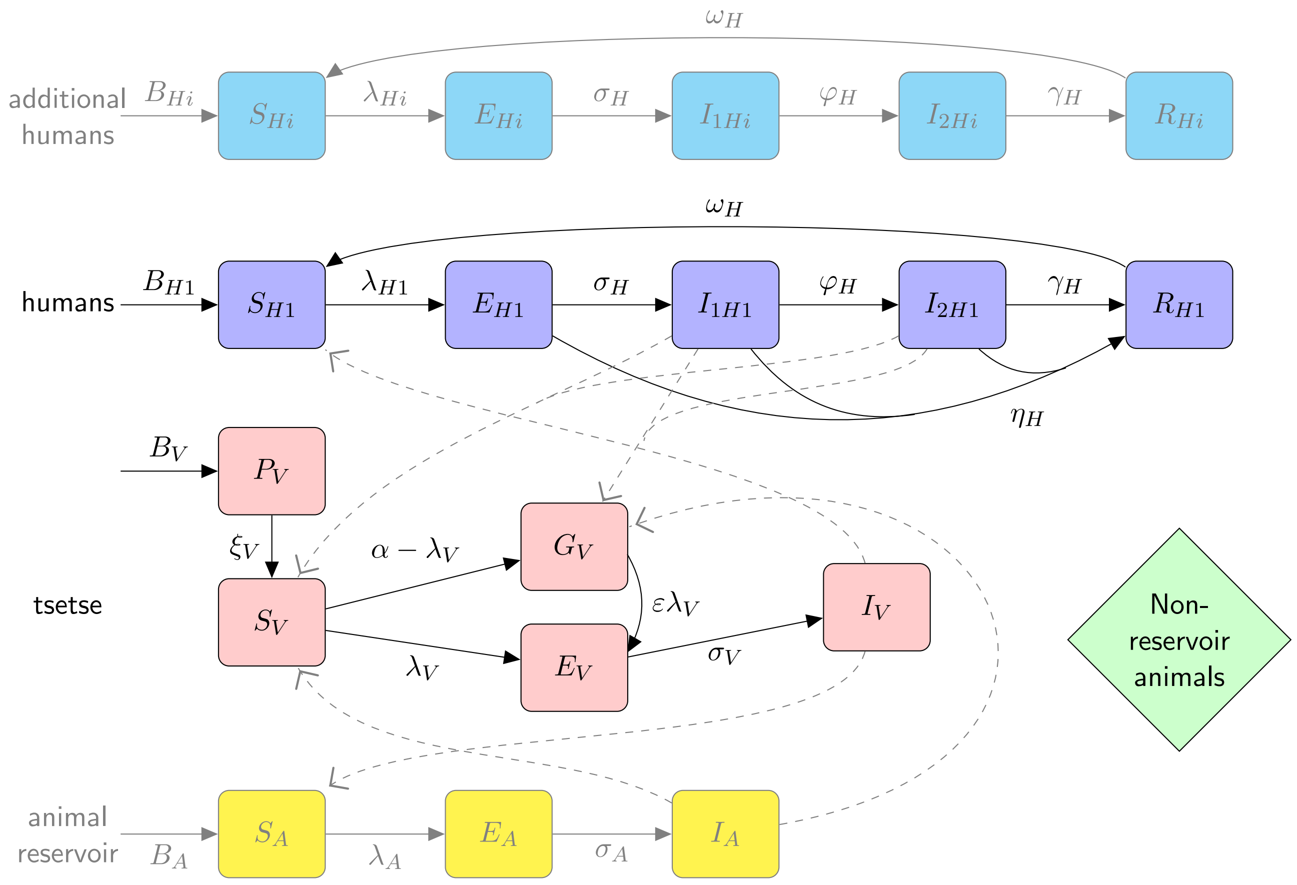


Figure S3: Multi-host model of HAT with one host species able to confer HAT (humans), a further non-reservoir species (others) and tsetse. Human hosts follow the progression which includes an infectious stage I disease, $I_{H1}$, infectious stage II disease, $I_{H2}$, and a non-infectious (due to hospitalisation) disease, $R_1$. $P_V$ are pupal stage tsetse which emerge into unfed adults. Unfed tsetse are susceptible, $S_V$, and following a blood-meal become either exposed, $E_V$, or have reduce susceptibility to the trypanosomes, $G_V$. Tsetse select their blood-meal from one of the two host species dependant upon innate feeding preference, $s$, and relative host abundance, $k$. Any blood-meals taken upon "other" hosts do not result in infection. The transmission of infection between humans/tsetse and reservoirs/tsetse is shown by grey paths. This figure is adapted from the original model schematic [S11].

$$
\text{Humans}\begin{cases}
\frac{\mathrm{d}S_{Hi}}{\mathrm{d}t} &= \mu_H N_{Hi} + \omega_H R_{Hi} - \alpha m_{\text{eff}} f_i \frac{S_{Hi}}{N_{Hi}} I_V - \mu_H S_{Hi} \\
\frac{\mathrm{d}E_{Hi}}{\mathrm{d}t} &= \alpha m_{\text{eff}} f_i \frac{S_{Hi}}{N_{Hi}} I_V - (\sigma_H + \mu_H) E_{Hi} \\
\frac{\mathrm{d}I_{1Hi}}{\mathrm{d}t} &= \sigma_H E_{Hi} - (\varphi_H + \mu_H) I_{1Hi} \\
\frac{\mathrm{d}I_{2Hi}}{\mathrm{d}t} &= \varphi_H I_{1Hi} - (\gamma_H + \mu_H) I_{2Hi} \\
\frac{\mathrm{d}R_{Hi}}{\mathrm{d}t} &= \gamma_H I_{2Hi} - (\omega_H + \mu_H) R_{Hi}
\end{cases}
$$

$$
\text{Animals}\begin{cases}
\frac{\mathrm{d}S_A}{\mathrm{d}t} &= \mu_A N_A - \alpha m_{\text{eff}} f_A \frac{S_A}{N_A} I_V - \mu_A S_A \\
\frac{\mathrm{d}E_A}{\mathrm{d}t} &= \alpha m_{\text{eff}} f_A \frac{S_A}{N_A} I_V - (\sigma_A + \mu_A) E_A \\
\frac{\mathrm{d}I_A}{\mathrm{d}t} &= \sigma_A E_A - \mu_A I_A
\end{cases}
$$

$$
\text{Tsetse}\begin{cases}
\frac{\mathrm{d}P_V}{\mathrm{d}t} &= B_V N_V - (\xi_V + \frac{P_V}{K}) P_V \\
\frac{\mathrm{d}S_V}{\mathrm{d}t} &= \xi_V \mathbb{P}(\text{survive pupal stage}) P_V - \alpha p_V \big( \sum_i f_i \frac{(I_{1Hi} + I_{2Hi})}{N_{Hi}} + f_A \frac{I_A}{N_A} \big) S_V \\
&\quad -\mu_V S_V \\
\frac{\mathrm{d}E_{1V}}{\mathrm{d}t} &= \alpha (1 - f_T(t)) p_V \big( \sum_i f_i \frac{(I_{1Hi} + I_{2Hi})}{N_{Hi}} + f_A \frac{I_A}{N_A} \big) (S_V + \varepsilon G_V) \\
&\quad -(3\sigma_V + \mu_V + \alpha f_T(t)) E_{1V} \\
\frac{\mathrm{d}E_{2V}}{\mathrm{d}t} &= 3\sigma_V E_{1V} - (3\sigma_V + \mu_V + \alpha f_T(t)) E_{2V} \\
\frac{\mathrm{d}E_{3V}}{\mathrm{d}t} &= 3\sigma_V E_{2V} - (3\sigma_V + \mu_V + \alpha f_T(t)) E_{3V} \\
\frac{\mathrm{d}I_V}{\mathrm{d}t} &= 3\sigma_V E_{3V} - (\mu_V + \alpha f_T(t)) I_V \\
\frac{\mathrm{d}G_V}{\mathrm{d}t} &= \alpha (1 - f_T(t)) \Big( - p_V \big( \sum_i f_i \frac{(I_{1Hi} + I_{2Hi})}{N_{Hi}} + f_A \frac{I_A}{N_A} \big) \Big) S_V \\
&\quad -\alpha \Big( f_T(t) + (1 - f_T(t)) p_V \varepsilon \big( \sum_i f_i \frac{(I_{1Hi} + I_{2Hi})}{N_{Hi}} + f_A \frac{I_A}{N_A} \big) G_V \big) \\
&\qquad -\mu_V G_V
\end{cases}
\tag{S1.5}
$$

N.B. Here the $N_H = \sum_i N_{Hi}$ and the actual number of vectors is $S_V, E_{1V}, E_{2V}, E_{3V}$ and $I_V$ multiplied by $N_V/N_H$.

$\sum_i f_i = f_H$ i.e. the total proportion of tsetse bites taken on humans. $s_i$ is the relative availability/attractiveness of different host types, so for the 4 different humans types (low/random participant, high/random, low/non-participant, high/non), where high risk humans are $r$-fold more likely to receive bites, $s = (1, r, 1, r)$. The $f_i$'s are calculated using $f_i = \frac{s_i N_{Hi}}{\sum_j s_j N_{Hj}}$.

Table S2: Parameter notation and values used in this analysis, following Rock *et al* [S11]

| Notation | Description | Value | Source |
|---|---|---|---|
| $\mu_H$ | Natural human mortality rate | 5.4795 $\times 10^{-5}$ days$^{-1}$ | [45] |
| $B_H$ | Human birth rate | $= \mu_H N_H$ | - |
| $\sigma_H$ | Human incubation rate | 0.0833 days$^{-1}$ | [S12] |
| $\varphi_H$ | Stage 1 to 2 progression rate | 0.0019 days$^{-1}$ | [S4, S5] |
| $\gamma_H$ | Treatment rate from stage 2 | 0.006 days$^{-1}$ | Assumed |
| | Frequency of screening | Annual | - |
| | Active screen diagnostic algorithm sensitivity | 0.91 | Averaged from [S3] |
| | Active screen diagnostic algorithm sensitivity | 0.999 | Averaged from [S3] |
| | Treatment compliance | 1 | Assumed |
| $\eta_H$ | Pulsed active screening | | |
| $\omega_H$ | Recovery rate | 0.006 days$^{-1}$ | [S9] |
| $N_H$ | Total human population size | 291567 | [S1, S2] |
| $k_1$ | Proportion of low-risk, randomly participating individuals | - | Fitted |
| $m$ | Relative tsetse density | $= N_V/N_H$ | - |
| $\mu_V$ | Tsetse mortality rate | 0.03 days$^{-1}$ | [S12] |
| $\alpha$ | Tsetse bite rate | 0.333 days$^{-1}$ | [S13] |
| $\sigma_V$ | Tsetse incubation rate | 0.034 days$^{-1}$ | [S7, S10] |
| $p_V$ | Probability of tsetse infection per single infective bite | 0.065 | [S11] |
| $p_H$ | Probability of human infection per single infective bite | - | Fitted |
| $m_{eff}$ | Effective tsetse density | $= mp_H$ | Fitted |
| $\varepsilon$ | Reduced non-teneral susceptibility | 0.05 | [S11] |
| $f_H$ | Proportion of blood-meal on humans | 0.09 | [S6] |
| $r$ | relative risk of taken on high-risk humans compared to low-risk | - | Fitted |
| $\mu_A$ | | 0.0014 days$^{-1}$ | Assumed |
| $B_A$ | Reservoir animal birth rate | $= \mu_A N_A$ | - |
| $\sigma_A$ | Reservoir animal incubation rate | 0.0833 days$^{-1}$ | [S12] |
| $f_A$ | Proportion of blood-meals on reservoir animals | - | Fitted |
| $N_A$ | Reservoir animal population size | - | Fitted |
| $p_A$ | Probability of reservoir animal infection per single infective bite | - | Fitted |

## S2 Model output

The compartmental ODE model is simulated to compute the disease dynamics in humans, animals and tsetse (see Fig. S4). The total annual passive reported cases for year, $T$ is calculated by integrating over the new hospitalisations from self-presentation multiplied by the reporting parameter, $u$, to compensate for underreporting of passive cases:

$$P_M = u \sum_i \int_T^{T+1} \gamma_H I_{2Hi}(t)\,\mathrm{d}t \tag{S2.1}$$

where $i \in$ all human types), whereas the active number of reported cases is given as:

$$\begin{aligned} A_M \;=\; & \textstyle\sum_j \text{ proportion screened} \\ & \times \text{ test sensitivity} \\ & \quad \times \text{ compliance} \\ & \quad\quad \times (I_{1Hj}(T) + I_{2Hj}(T)) \\ + \; & \text{proportion screened} \\ & \times \ (1 - \text{test specificity}) \\ & \quad \times \text{ compliance} \\ & \quad\quad \times (N_{Hj}(T) - I_{1Hj}(T) + I_{2Hj}(T)) \end{aligned} \tag{S2.2}$$

where $j \in$ random participants. The number of reported cases seen under the model is also shown in Fig S4.

Previous model fitting to data from 2000–2012 using MCMC [S11] already provided replicates from which to project forwards to establish the expected elimination and elimination as a public health problem years under the different strategies. The results presented here took 1000 samples from the posterior parameter distribution that was previously computed and simulated forwards under the deterministic model with each strategy, therefore the observed variation is due to parameter uncertainty rather than stochastic effects.

## S3 Additional results

The key results are presented in the main text, these consist of six strategies including three with and three without vector control. The main text strategies represent mean screening levels (30%) and moderate vector population reductions (60%). Other variations on these strategies are shown here which include the maximum level of active screening achieved between 2000–2012 (53%) and high-efficacy vector control (90% reduction) starting in either 2017 (as per the main text) or in 2018. The eighteen strategies are given in Table S3. Table S4 and Figure S5 show the mean and 95% credible intervals for the full elimination year under the eighteen proposed strategies. Likewise Table S5 and Figure S6 show the same information for the year of elimination as a public health problem. The number of new cases averted compared to mean screening alone (Strategy 1) is presented in Table S6.

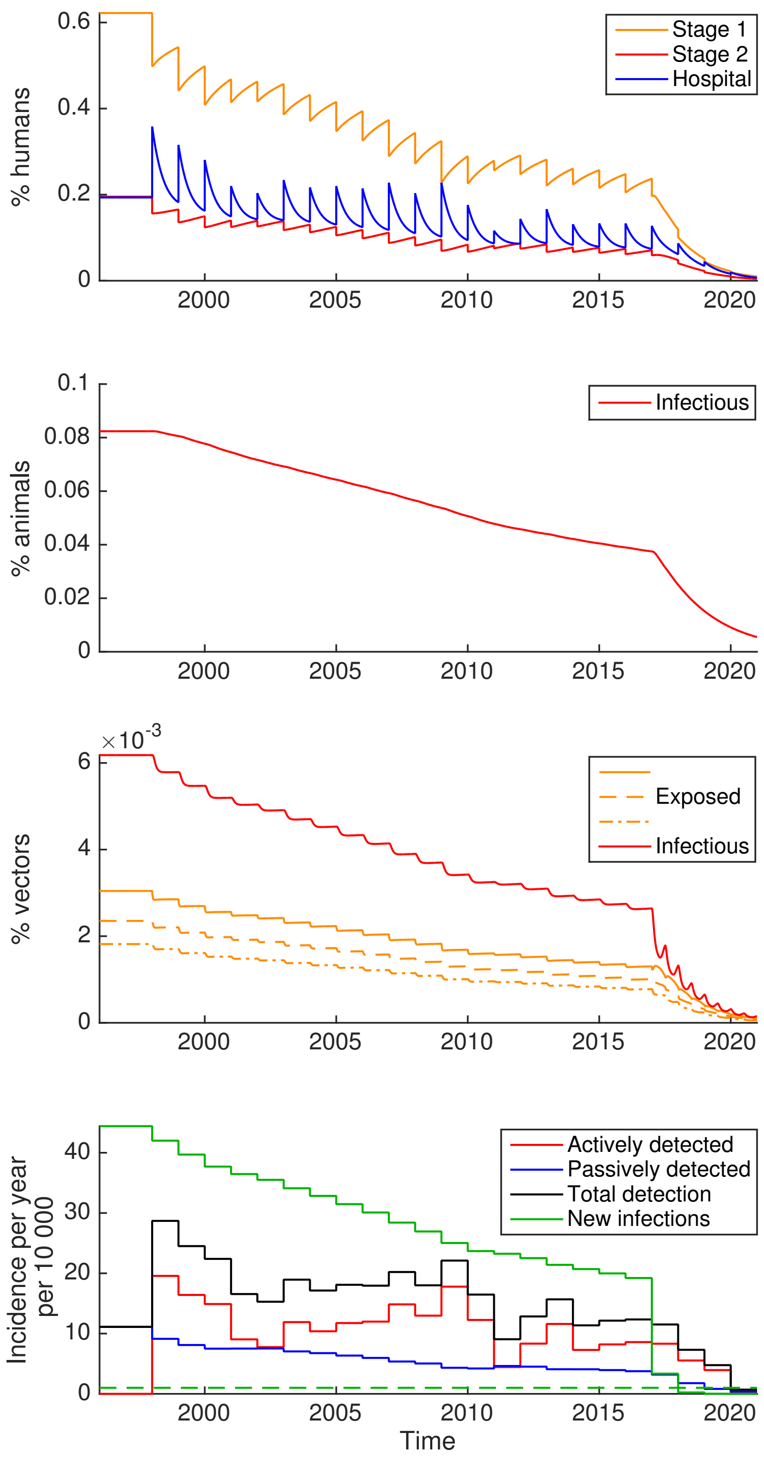


Figure S4: Example disease dynamics of the human, animal, tsetse model. The top 3 graphs show the continuous disease dynamics generated by the ODE model, with active, pulsed screening taking place annually from 1998 and a passive reporting level of $u = 0.32$. Additionally vector control with a 90% reduction in tsetse after one year is started in 2017. The bottom graph shows the incidence per year per 10,000 which is computed after obtaining the solutions to the ODE (see (S2.1) and (S2.2)).

Table S3: **Variations on key intervention strategies under consideration**

| | **Recruited to screening** | | | **Screening %** | | **VC start** | | | **Tsetse reduction** | | |
|---|---|---|---|---|---|---|---|---|---|---|---|
| Strategy | Low-risk only | Equal | High-risk first | Mean | Max | Never | 2017 | 2018 | 0% | 60% | 90% |
| 1 - Screen low-risk | X | | | X | | X | | | X | | |
| 2 | X | | | | X | X | | | X | | |
| 3 - Screen equally | | X | | X | | X | | | X | | |
| 4 | | X | | | X | X | | | X | | |
| 5 - Screen high-risk | | | X | X | | X | | | X | | |
| 6 | | | X | | X | X | | | X | | |
| 7 - Screen low-risk + VC | X | | | X | | | X | | | X | X* |
| 8 | X | | | | X | | X | | | X | X |
| 9 - Screen equally + VC | | X | | X | | | X | | | X | X* |
| 10 | | X | | | X | | X | | | X | X |
| 11 - Screen high-risk + VC | | | X | X | | | X | | | X | X* |
| 12 | | | X | | X | | X | | | X | X |
| 13 | X | | | X | | | | X | | X | X |
| 14 | X | | | | X | | | X | | X | X |
| 15 | | X | | X | | | | X | | X | X |
| 16 | | X | | | X | | | X | | X | X |
| 17 | | | X | X | | | | X | | X | X |
| 18 | | | X | | X | | | X | | X | X |

* Not shown in main text but included here

This table presents the proposed screening and vector control strategies including the named strategies (1, 3, 5, 7, 9 and 11) which are those presented in the main text. For strategies with vector control it is assumed that either 60% or 90% reductions in tsetse density are achieved and both variants are presented alongside strategies 7–18.

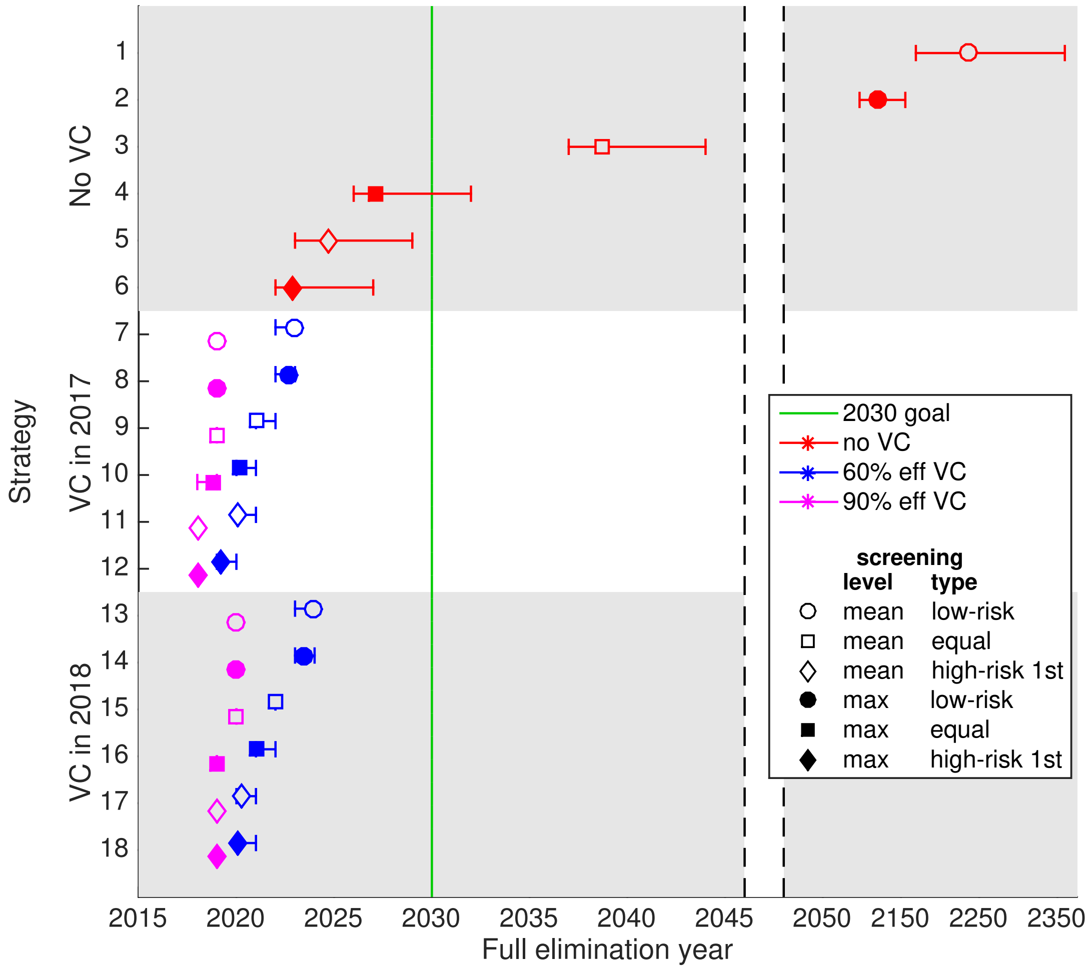


Figure S5: **Impact of other possible strategies on elimination** The figure shows the predicted year in which transmission drops below 1 case per 100,000 under eighteen different strategies and compares this to the target year of 2030.

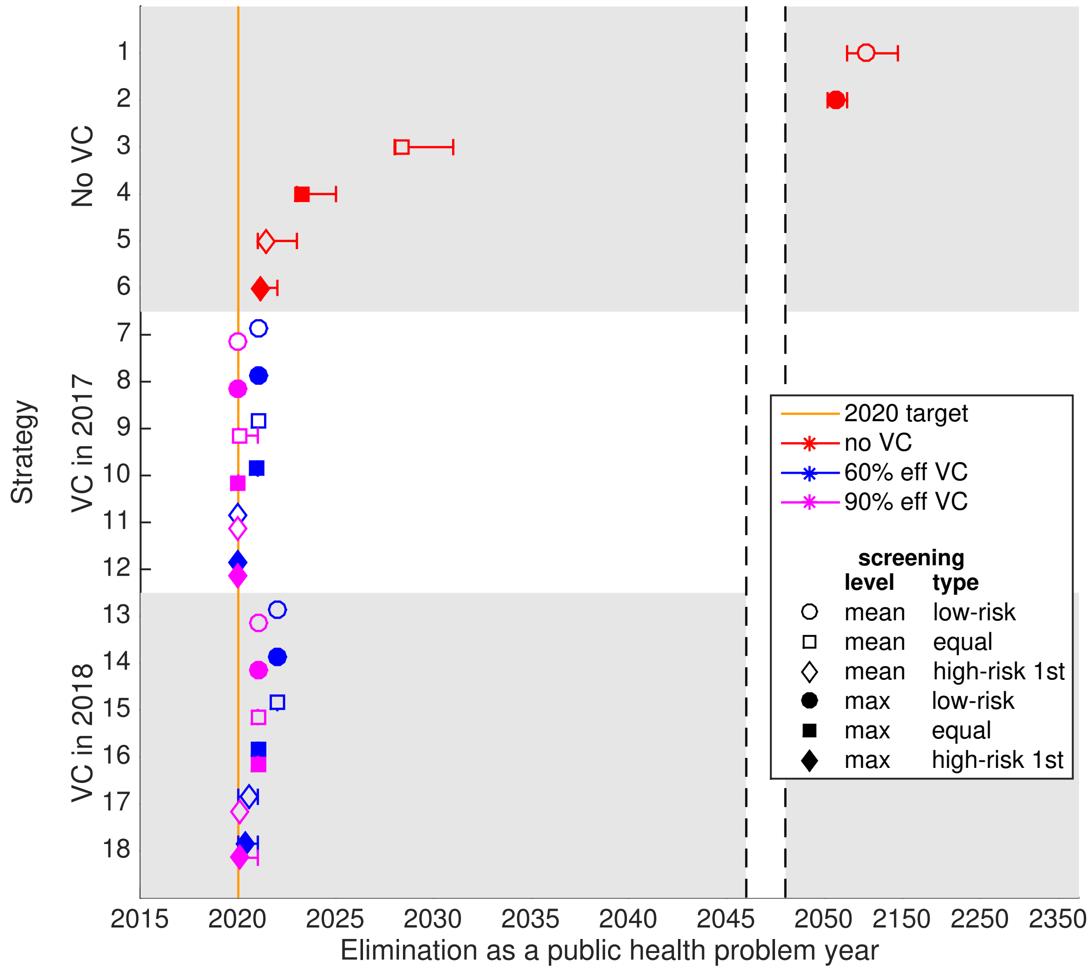


Figure S6: **Impact of other possible strategies on elimination as a public health problem** The figure shows the predicted year in which the reported cases drop below 1 case per 10,000 under eighteen different strategies. The results are compared to the year 2020 to see if it is possible that these regions could be amongst the foci that achieve the elimination as a public health problem target within this timeframe.

Table S4: **Projected full elimination year for all strategy variants.**

| | **Recruited to screening** | | | **Screening %** | | **VC start** | | | **Tsetse reduction** | | |
|---|---|---|---|---|---|---|---|---|---|---|---|
| | Low-risk only | Equal | High-risk first | Mean | Max | Never | 2017 | 2018 | 0% | 60% | 90% |
| 1 | X | | | X | | X | | | 2236.2 [2169, 2360] | | |
| 2 | X | | | | X | X | | | 2120.7 [2097, 2156] | | |
| 3 | | X | | X | | X | | | 2038.7 [2037, 2044] | | |
| 4 | | X | | | X | X | | | 2027.2 [2026, 2032] | | |
| 5 | | | X | X | | X | | | 2024.7 [2023, 2029] | | |
| 6 | | | X | | X | X | | | 2022.8 [2022, 2027] | | |
| 7 | X | | | X | | | X | | | 2022.9 [2022, 2023] | 2019.0 [2019, 2019] |
| 8 | X | | | | X | | X | | | 2022.7 [2022, 2023] | 2019.0 [2019, 2019] |
| 9 | | X | | X | | | X | | | 2021.1 [2021, 2022] | 2019.0 [2019, 2019] |
| 10 | | X | | | X | | X | | | 2020.1 [2020, 2021] | 2018.8 [2018, 2019] |
| 11 | | | X | X | | | X | | | 2020.1 [2020, 2021] | 2018.0 [2018, 2018] |
| 12 | | | X | | X | | X | | | 2019.2 [2019, 2020] | 2018.0 [2018, 2018] |
| 13 | X | | | X | | | | X | | 2023.9 [2023, 2024] | 2020.0 [2020, 2020] |
| 14 | X | | | | X | | | X | | 2023.4 [2023, 2024] | 2020.0 [2020, 2020] |
| 15 | | X | | X | | | | X | | 2022.0 [2022, 2022] | 2020.0 [2020, 2020] |
| 16 | | X | | | X | | | X | | 2021.0 [2021, 2022] | 2019.0 [2019, 2019] |
| 17 | | | X | X | | | | X | | 2020.2 [2020, 2021] | 2019.0 [2019, 2019] |
| 18 | | | X | | X | | | X | | 2020.1 [2020, 2021] | 2019.0 [2019, 2019] |

Expected year of HAT elimination (less than 1 new transmission per 10,000) in Yasa Bonga and Mosango health zones under each of the proposed strategies with either 60% or 90% reductions in tsetse density achieved. 95% credible intervals are given in brackets. This combines results of both Models 4 and 7 (with and without animal reservoirs).

Table S5: **Elimination as a public health problem year projections for different strategies.**

| | **Recruited to screening** | | | **Screening %** | | **VC start** | | | **Tsetse reduction** | | |
|---|---|---|---|---|---|---|---|---|---|---|---|
| | Low-risk only | Equal | High-risk first | Mean | Max | Never | 2017 | 2018 | 0% | 60% | 90% |
| 1 | X | | | X | | X | | | 2103.5 [2079, 2144] | | |
| 2 | X | | | | X | X | | | 2064.0 [2054, 2079] | | |
| 3 | | X | | X | | X | | | 2028.4 [2028, 2031] | | |
| 4 | | X | | | X | X | | | 2023.3 [2023, 2025] | | |
| 5 | | | X | X | | X | | | 2021.4 [2021, 2023] | | |
| 6 | | | X | | X | X | | | 2021.1 [2021, 2022] | | |
| 7 | X | | | X | | | X | | | 2021.0 [2021, 2021] | 2020.0 [2020, 2020] |
| 8 | X | | | | X | | X | | | 2021.0 [2021, 2021] | 2020.0 [2020, 2020] |
| 9 | | X | | X | | | X | | | 2021.0 [2021, 2021] | 2020.1 [2020, 2021] |
| 10 | | X | | | X | | X | | | 2021.0 [2021, 2021] | 2020.0 [2020, 2020] |
| 11 | | | X | X | | | X | | | 2020.0 [2020, 2020] | 2020.0 [2020, 2020] |
| 12 | | | X | | X | | X | | | 2020.0 [2020, 2020] | 2020.0 [2020, 2020] |
| 13 | X | | | X | | | | X | | 2022.0 [2022, 2022] | 2021.0 [2021, 2021] |
| 14 | X | | | | X | | | X | | 2022.0 [2022, 2022] | 2021.0 [2021, 2021] |
| 15 | | X | | X | | | | X | | 2022.0 [2022, 2022] | 2021.0 [2021, 2021] |
| 16 | | X | | | X | | | X | | 2021.0 [2021, 2021] | 2021.0 [2021, 2021] |
| 17 | | | X | X | | | | X | | 2020.6 [2020, 2021] | 2020.0 [2020, 2020] |
| 18 | | | X | | X | | | X | | 2020.4 [2020, 2021] | 2020.0 [2020, 2021] |

Expected year of HAT elimination as a public health problem (less than 1 reported case per 10,000) in Yasa Bonga and Mosango health zones under each of the proposed strategies with either 60% or 90% reductions in tsetse density achieved. 95% credible intervals are given in brackets. This combines results of both Models 4 and 7 (with and without animal reservoirs).

Table S6: **Projected new cases averted between 2017 and 2030 for all strategy variants.**

| | **Recruited to screening** | | | **Screening %** | | **VC start** | | | **Tsetse reduction** | | |
|---|---|---|---|---|---|---|---|---|---|---|---|
| | Low-risk only | Equal | High-risk first | Mean | Max | Never | 2017 | 2018 | 0% | 60% | 90% |
| 1 | X | | | X | | X | | | 0 [0, 0] | | |
| 2 | X | | | | X | X | | | 1410 [1313, 1496] | | |
| 3 | | X | | X | | X | | | 4979 [4003, 6032] | | |
| 4 | | X | | | X | X | | | 6320 [5215, 7540] | | |
| 5 | | | X | X | | X | | | 6808 [5616, 8133] | | |
| 6 | | | X | | X | X | | | 6948 [5774, 8264] | | |
| 7 | X | | | X | | | X | | | 6880 [5756, 8163] | 7192 [6027, 8523] |
| 8 | X | | | | X | | X | | | 6911 [5788, 8191] | 7200 [6035, 8531] |
| 9 | | X | | X | | | X | | | 6992 [5850, 8288] | 7210 [6042, 8543] |
| 10 | | X | | | X | | X | | | 7087 [5933, 8392] | 7230 [6061, 8564] |
| 11 | | | X | X | | | X | | | 7161 [5992, 8485] | 7248 [6075, 8585] |
| 12 | | | X | | X | | X | | | 7186 [6018, 8509] | 7255 [6082, 8591] |
| 13 | X | | | X | | | | X | | 6250 [5212, 7438] | 6551 [5470, 7787] |
| 14 | X | | | | X | | | X | | 6332 [5294, 7516] | 6604 [5522, 7839] |
| 15 | | X | | X | | | | X | | 6532 [5445, 7763] | 6700 [5593, 7956] |
| 16 | | X | | | X | | | X | | 6776 [5665, 8038] | 6860 [5736, 8133] |
| 17 | | | X | X | | | | X | | 6984 [5831, 8297] | 7024 [5867, 8335] |
| 18 | | | X | | X | | | X | | 7043 [5891, 8353] | 7068 [5912, 8378] |

Predicted number of new cases averted between 2017 and 2030 for each strategy compared to the base case (Strategy 1, with no vector control and screening at 29.9% of the low-risk population).

## References

[S1] Action Contre la Faim International. Enquête Nutritionnelle Anthropométrique: Zone de Santé de Yasa Bonga. Technical report, 2009.

[S2] Action Contre la Faim International. Dépistage nutritionnel rapide: Zones de Santé rurale de Mosango. Technical report, 2012.

[S3] F Checchi, F Chappuis, Unni Karunakara, Gerardo Priotto, and D Chandramohan. Accuracy of Five Algorithms to Diagnose Gambiense Human African Trypanosomiasis. *PLoS Neglected Tropical Diseases*, 5(7):e1233–15, July 2011.

[S4] F Checchi, J A N Filipe, M P Barrett, and D Chandramohan. The natural progression of Gambiense sleeping sickness: What is the evidence? *PLoS Neglected Tropical Diseases*, 2(12):e303, December 2008.

[S5] F Checchi, S Funk, D Chandramohan, D T Haydon, and F Chappuis. Updated estimate of the duration of the meningo-encephalitic stage in gambiense human African trypanosomiasis. *BMC Research Notes*, 8(1):292, July 2015.

[S6] P-H Clausen, I Adeyemi, B Bauer, M Breloeer, F Salchow, and C Staak. Host preferences of tsetse (Diptera: Glossinidae) based on bloodmeal identifications. *Medical and Veterinary Entomology*, 12(2):169–180, March 1998.

[S7] S Davis, S Aksoy, and A P Galvani. A global sensitivity analysis for African sleeping sickness. *Parasitology*, 138(04):516–526, November 2010.

[S8] J W Hargrove, R Ouifki, and J E Ameh. A general model for mortality in adult tsetse (*Glossina* spp.). *Medical and Veterinary Entomology*, 25(4):385–394, March 2011.

[S9] A Mpanya, D Hendrickx, M Vuna, A Kanyinda, C Lumbala, V Tshilombo, P Mitashi, O Luboya, V Kande, M Boelaert, P Lefèvre, and P Lutumba. Should I Get Screened for Sleeping Sickness? A Qualitative Study in Kasai Province, Democratic Republic of Congo. *PLoS Neglected Tropical Diseases*, 6(1):e1467, January 2012.

[S10] S Ravel, P Grebaut, D Cuisance, and G Cuny. Monitoring the developmental status of Trypanosoma brucei gambiense in the tsetse fly by means of PCR analysis of anal and saliva drops. *Acta Tropica*, 88(2):161–165, October 2003.

[S11] K S Rock, S J Torr, C Lumbala, and M J Keeling. Quantitative evaluation of the strategy to eliminate human African trypanosomiasis in the Democratic Republic of Congo. *Parasites & Vectors*, 8(1):1–13, October 2015.

[S12] D J Rogers. A general model for the African trypanosomiases. *Parasitology*, 97:193–212, 1988.

[S13] WHO. Control and surveillance of human African trypanosomiasis. Technical Report 984, November 2013.
